# Using automated activity monitoring to detect resumption of cyclicity in early lactation—Meta-analysis

**DOI:** 10.3168/jdsc.2025-0785

**Published:** 2025-07-23

**Authors:** S. Borchardt, T.A. Burnett, T.C. Bruinjé, A.M.L. Madureira

**Affiliations:** 1 Farm Animal Clinic, Division for Ruminants and Camelids, Unit for Reproduction Medicine and Udder Health, School of Veterinary Medicine, Freie Universitaet Berlin, 14163 Berlin, Germany; 2 University of Guelph, Ridgetown Campus, Ridgetown, ON, Canada N0P 2C0; 3 Department of Dairy and Food Science, South Dakota State University, Brookings, SD 57007; 4 Animal Science Department, Michigan State University, East Lansing, MI 48824

## Abstract

This meta-analysis aimed to evaluate the efficacy of automated activity monitoring (AAM) in detecting estrous expression and ovulatory status in cows during the voluntary waiting period (VWP). A comprehensive literature search was conducted in PubMed, ScienceDirect, and Google Scholar using specific search terms. Inclusion criteria focused on studies that assessed estrous expression within the VWP using modern AAM systems alongside blood progesterone (P4) measurements. Four manuscripts involving 2,198 cows were included. Data extraction was performed by a single investigator and validated by a coauthor. The analysis considered estrous expression and ovulatory status determined through serial blood P4 measurements. Cows were classified based on P4 concentrations and estrus alerts into true positive, false positive, true negative, and false negative categories. Statistical analyses were conducted using MedCalc, incorporating 7 experimental groups from the selected manuscripts. The pooled proportion of ovulatory cows with P4 ≥1 ng/mL by 49 DIM (in most studies) was 79.8% (95% CI: 74.9%–84.3%), with significant heterogeneity (I^2^ = 86.1%). The proportion of cows with estrous expression detected by AAM by 60 DIM (in most studies) was 64.0% (95% CI: 48.3%–78.3%), also showing significant heterogeneity (I^2^ = 98.1%). Sensitivity and specificity of AAM systems to identify ovulatory cows were 70.3% (95% CI: 55.1%–83.4%) and 60.0% (95% CI: 42.5%–76.3%), respectively, both with significant heterogeneity. Positive predictive value was 88.1% (95% CI: 84.9%–91.0%), and negative predictive value was 35.3% (95% CI: 26.3%–44.7%), indicating variability among experimental groups. The findings suggest that although AAM systems show promise in confirming ovulation in early lactation, the lack of estrus detection by AAM does not confirm anovulatory status. The heterogeneity in the data suggests there might be inconsistencies in the precision or configuration of the AAM systems across farms and studies.

## Highlights

• A meta-analysis was conducted, including 4 manuscripts and 2,198 cows.
• 79.8% of cows were ovulatory, and 64.0% exhibited estrous expression.
• Automated activity monitors were accurate in identifying ovulatory cows.
• Automated activity monitors failed to identify anovulatory cows.

## Graphical Abstract

**Figure:**
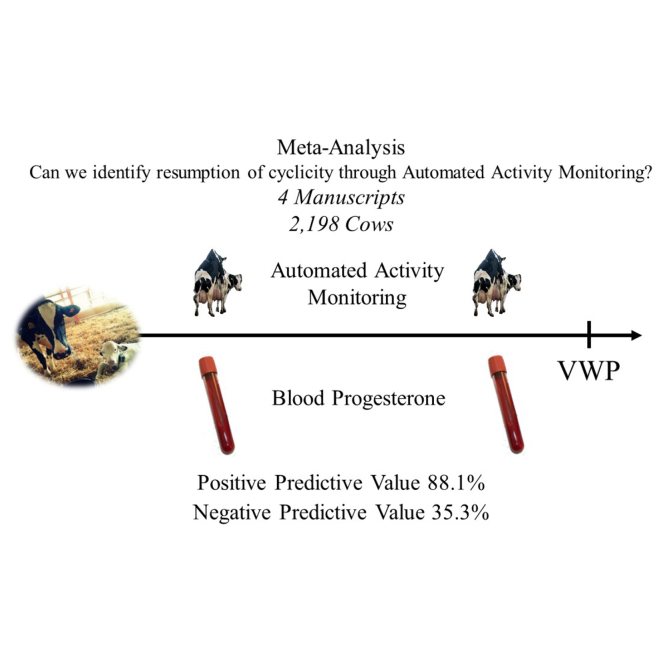
**Summary:** The objective of this meta-analysis was to evaluate the efficacy of automated activity monitors (AAM) in identifying ovulation and, consequently, the resumption of ovarian cyclicity in lactating dairy cows in early lactation. Inclusion criteria focused on studies that assessed estrous expression within the voluntary waiting period (VWP) using modern AAM systems alongside blood progesterone measurements. Four manuscripts with 7 experimental groups involving 2,198 cows were included. Cows were classified based on progesterone concentrations and estrus alerts into true positive, false positive, true negative, and false negative categories. The majority of cows (79.8%) ovulated. Sixty-four percent of the cows had an estrus alert. Sensitivity and specificity of AAM systems to identify ovulatory cows were 70.3% and 60.0%, respectively. Positive predictive value was 88.1%, and negative predictive value was 35.3%. The findings suggest that AAM systems show promise in confirming ovulation in early lactation. The lack of estrus detection by AAM does not confirm anovulatory status.

Resumption of ovarian cyclicity postpartum has a major impact on reproductive performance in dairy cattle (Dubuc et al., 2012). It has been estimated that one-quarter of lactating Holstein cows are anovular at the beginning of the breeding period using data from multiple large observational studies (Walsh et al., 2007; Santos et al., 2009; Dubuc et al., 2012; Pinedo et al., 2020). In these studies, anovulation was determined either by transrectal ultrasound examination of the ovaries or blood/milk progesterone (**P4**). Although considered as the gold standard, this is labor intensive, requiring multiple examinations (Lucy, 2019). Consequently, routine monitoring of ovarian cyclicity status has not been adopted on commercial dairy farms. The adoption of automated activity monitoring (**AAM**) systems provides an opportunity to collect information on estrous expression within the voluntary waiting period (**VWP**) as a proxy for resumption of ovarian cyclicity without additional effort (Bretzinger et al., 2024). It has been shown in multiple studies that cows with estrous expression within the VWP have a greater insemination rate, greater pregnancy per AI (**P/AI**) at first service, and reduced median days open compared with cows that show no estrus within the VWP (Borchardt et al., 2021; Bretzinger et al., 2023; Bruinjé and LeBlanc, 2024). Therefore, the occurrence of estrus within the VWP can be used to identify subgroups of cows with superior or inferior predicted reproductive performance. Several studies have used this concept for a targeted reproductive management approach where cows with estrous expression within the VWP were primarily bred after estrus detection during the breeding period, whereas cows without estrous expression within the VWP were enrolled into TAI protocols (Rial et al., 2022; Gonzalez et al., 2023; Laplacette et al., 2024). The goal of these protocols, such as Double-Ovsynch or Ovsynch with P4 supplementation, was to optimize reproductive performance in the subgroup of anovular cows. To optimize targeted reproductive management based on estrus activity within the VWP, it is crucial to understand the accuracy and variability of AAM systems in identifying ovulatory or anovulatory cows.

Individual studies often encompass a limited number of cows or herds, under similar management practices, climatic conditions, AAM system, and genetic background, which might limit external validity of findings (Tempelman, 2009). To address this challenge, a meta-analysis was conducted to evaluate the efficacy of AAM systems in identifying ovulation and, consequently, the resumption of ovarian cyclicity in lactating dairy cows in early lactation. Automated activity monitors are widely used tools in reproductive management and accurate in detecting estrus and ovulation during the breeding period (Roelofs et al., 2017; Adriaens et al., 2019). However, studies have shown a substantial proportion (35% to 75%) of cows with no estrous expression alerted by the AAM that had in fact ovulated (i.e., false negatives; Borchardt et al., 2024; Bretzinger et al., 2024; Laplacette et al., 2024). Therefore, we hypothesized that AAM systems would have acceptable accuracy in identifying ovulatory cows (>70% sensitivity), but not in detecting anovulatory cows.

The literature search was conducted in PubMed (http://www.ncbi.nlm.nih.gov/pubmed), ScienceDirect (http://www.sciencedirect.com), and Google Scholar (http://scholar.google.com) using the search terms “targeted reproductive management” OR “automated activity monitoring.” After eliminating duplicate articles, the literature search returned a total of 52 original research and review articles. Additional manuscripts were obtained directly from researchers in the field of reproductive management. Results from the literature search were assessed individually for the initial screening to be considered for the meta-analysis.

We considered only studies that assessed estrous expression within the VWP using an AAM system in conjunction with blood P4 measurements. To be eligible, studies needed to include animals that did not receive any hormonal intervention before the VWP, ensuring that the animals were showing estrus and ovulation spontaneously. Furthermore, studies needed to report data on estrous expression with the resultant ovulatory status, determined through serial blood P4 measurements, during the same observational period. Based on these criteria, the meta-analysis included a total of 4 manuscripts including 2,198 cows (Bruinjé et al., 2023; Bretzinger et al., 2024; Borchardt et al., 2024; Laplacette et al., 2024). Different AAM systems were used across the studies, and the information about these systems is presented in Table 1.

**Table 1:** Summary of the cow population, AAM system, and observation period reported in each of 7 studies reported in 4 manuscripts

Reference	No. of cows	AAM^1^ system	Location of the AAM system	Observation period for estrus, DIM	Timing of blood P4^2^ sample, DIM
Bruinjé et al., 2023	811	AfiAct	Leg	18–54	21/35/49
Borchardt et al., 2024	343	Nedap SmartTag	Neck	7–30	15/18/21/24/28/30
Bretzinger et al., 2024					
Exp. 1	192	Smartbow	Ear	7–60	21/35/49
Exp. 2	219	Heatime	Neck	7–60	21/35/49
Exp. 3	197	DelPro	Leg	7–60	21/35/49
Exp. 4	216	CowManager	Ear	7–60	21/35/49
Laplacette et al., 2024	220	SenseHub	Neck	15–49	34/46

1 AAM = automated activity monitoring; AfiAct, Afimilk, Kibbutz Afikim; Nedap SmartTag Neck, Nedap Livestock; Smartbow, Zoetis; Heatime, SCR Engineers Ltd.; DelPro, DeLaval; CowManager SensOor, Agis Automatisering; SenseHub Monitoring Neck Tag, Merck Animal Health Management.

2 P4 = progesterone.

Data extraction was performed by a single investigator (S. Borchardt) and validated by one coauthor (A. M. L. Madureira). For each study, recorded information included authors, year of publication, number of herds, VWP, AAM system, observation period for estrous expression within VWP, timing of blood P4 measurement, and threshold for P4 to determine ovulation. Relevant information is summarized in Table 1.

Resumption of ovarian cyclicity was considered when P4 concentration was ≥1 ng/mL at any collection day. Cows were considered anovular when P4 concentration was <1 ng/mL on all collection days. Progesterone concentrations were used as a gold standard to calculate the test characteristics of the AAM system. Cows were classified as true positive: cyclic and at least one estrus alert; false positive: anovular and at least one estrus alert; true negative: anovular and no estrus alerts; and false negative: cyclic and no estrus alert. Equations used to determine the precision (i.e., sensitivity, specificity, positive predictive value, and negative predictive value) of the AAM to detect cyclic cows are shown in Table 2.

**Table 2:** Equations used to determine the test characteristics of an automated activity monitor to identify cows that resumed ovarian cyclicity in early lactation based on plasma progesterone (P4) concentration

Test characteristic		Equation^1^
Sensitivity		TP/(TP + FN) × 100
Specificity		TN/(TN + FP) × 100
Positive predictive value		TP/(TP+FP) × 100
Negative predictive value		TN/(TN+FN) × 100

1 True positive (TP): P4 ≥1 ng/mL and at least one estrus alert. True negative (TN): P4 <1 ng/mL and no estrus alerts. False positive (FP): P4 <1 ng/mL and at least one estrus alert. False negative (FN): P4 ≥1 ng/mL and no estrus alerts.

The meta-analysis was conducted using MedCalc (version 23.2.1, MedCalc Software, Mariakerke, Belgium) as described elsewhere (Borchardt et al., 2017).

We included 7 experimental groups from 4 manuscripts with information on estrous expression and ovulatory status for 2,198 cows. To determine the proportion of ovulatory cows, the proportion of cows showing estrus, and the different test characteristics (i.e., sensitivity, specificity, positive predictive value, and negative predictive value), we calculated the pooled proportion from the 7 experimental groups. The pooled proportion is an overall estimate that considers both the individual estimate and its precision for the experimental group. The pooled proportions with their 95% CI under the fixed and the random effects model were compared. MedCalc uses a Freeman–Tukey transformation (Freeman and Tukey, 1950) to calculate the weighted summary proportion under the fixed and random effects model (DerSimonian and Laird, 1986). We reported the pooled proportion from the random effects model when significant heterogeneity was present (*P* ≤ 0.05). Heterogeneity (I^2^) is the percentage of observed total variation across studies that is due to real heterogeneity rather than chance. It is calculated as $I^{2}=100\&percnt;\times \frac{\left(Q-\mathrm{df}\right)}{Q},$ where *Q* = Cochran's heterogeneity statistic. Negative values of I^2^ are put equal to zero so that I^2^ lies between 0% and 100%. A value of 0% indicates no observed heterogeneity, and larger values represent increasing heterogeneity (Higgins et al., 2003).

The overall proportion of ovulatory cows within the observational period was 79.8% (95% CI: 74.9% to 84.3%). Significant heterogeneity existed (I^2^ = 86.1%; *P* = 0.001) among the experimental groups regarding ovulation. The individual proportion of ovulatory cows in the experimental groups ranged from 64.1% (95% CI: 56.8% to 70.8%; Borchardt et al., 2024) to 85.3% (95% CI: 82.7% to 87.7%; Bruinjé et al., 2023).

The overall proportion of cows with estrous expression within the observational period was 64.0% (95% CI: 48.3% to 78.3%). Significant heterogeneity existed (I^2^ = 98.1%; *P* = 0.001) among the experimental groups. The individual proportion of cows with estrous expression in the experimental groups ranged from 27.6% (95% CI: 21.4% to 34.5%; Borchardt et al., 2024) to 89.5% (95% CI: 84.7% to 93.2%; Bretzinger et al., 2024, experiment [**exp.**] 4).

The overall sensitivity of the AAM systems was 70.3% (95% CI: 55.1% to 83.4%; Figure 1), with significant heterogeneity (I^2^ = 97.6%; *P* = 0.001) among the experimental groups. The individual sensitivity in the experimental groups ranged from 34.1% (95% CI: 25.8% to 43.2%; Borchardt et al., 2024) to 92.3% (95% CI: 87.4% to 95.7%; Bretzinger et al., 2024, exp. 4).

**Figure 1:**
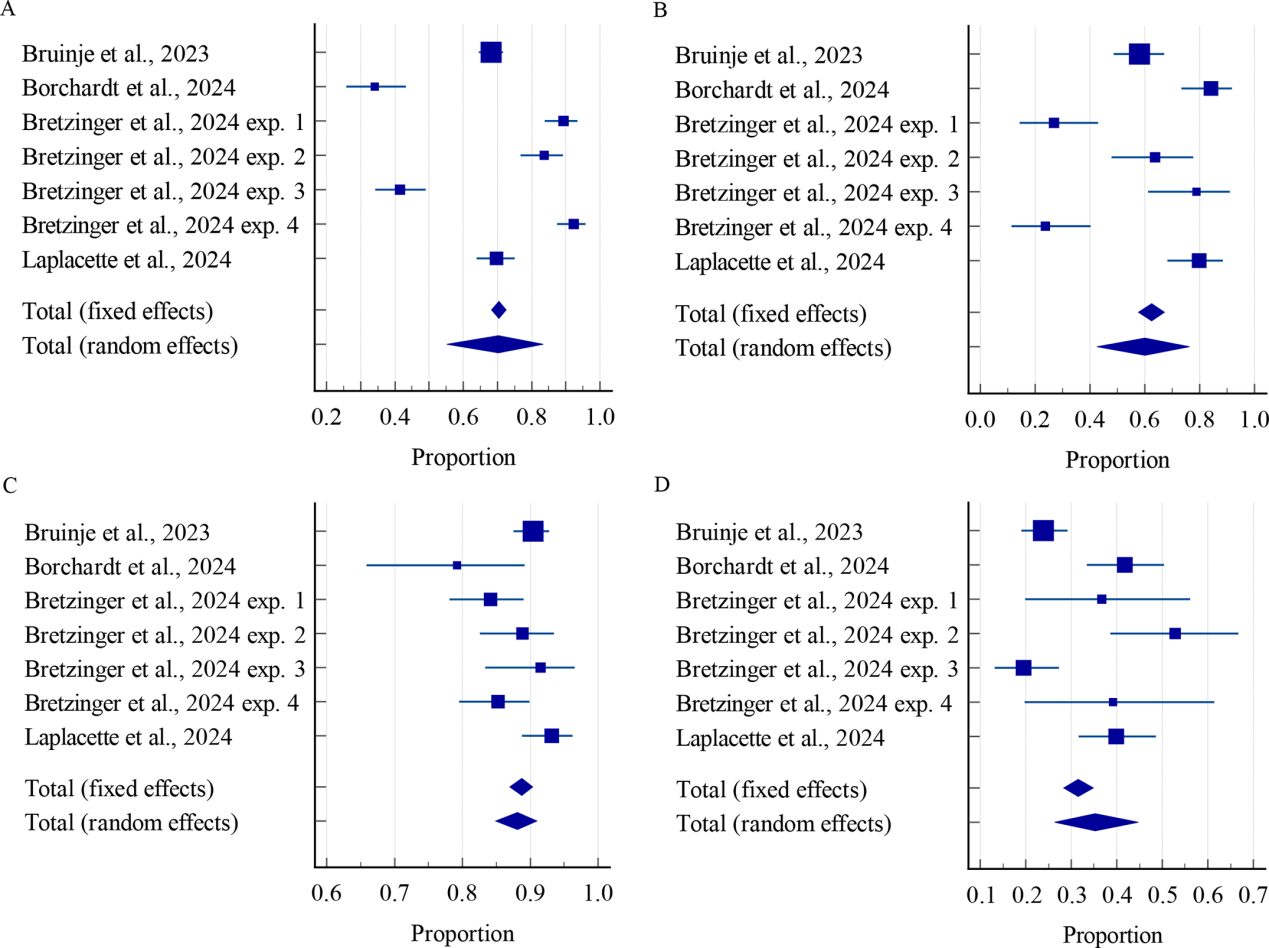
Different test characteristics (i.e., sensitivity: panel A, specificity: panel B, positive predictive value: panel C, negative predictive value: panel D) for automated activity monitoring systems to identify ovulatory cows in early lactation from 7 studies reported in 4 manuscripts with a total of 2,198 cows. The weight (solid square) and the 95% CI (whiskers) are depicted for each study. The overall pooled proportion (diamond) was summarized using either a fixed or a random effects model. References: Bruinjé et al., 2023; Borchardt et al., 2024; Bretzinger et al., 2024; Laplacette et al., 2024.

The overall specificity of the AAM systems was 60.0% (95% CI: 42.5% to 76.3%; Figure 1) with significant heterogeneity (I^2^ = 92.2%; *P* = 0.001) among the experimental groups. The individual specificity in the experimental groups ranged from 23.7% (95% CI: 11.4% to 40.2%; Bretzinger et al., 2024, exp. 4) to 84.1% (95% CI: 73.3% to 91.8%; Borchardt et al., 2024).

The overall proportion of cows that ovulated among cows with estrous expression (i.e., positive predictive value) was 88.1% (95% CI: 84.9% to 91.0%; Figure 1), with significant heterogeneity (I^2^ = 63.7%; *P* = 0.001) among the experimental groups. The individual positive predictive value in the experimental groups ranged from 79.2% (95% CI: 65.9% to 89.2%; Borchardt et al., 2024) to 93.2% (95% CI: 88.8% to 96.2%; Laplacette et al., 2024).

The overall proportion of cows that did not ovulated among cows without estrous expression (i.e., negative predictive value) was 35.3% (95% CI: 26.3% to 44.7%; Figure 1). Significant heterogeneity existed (I^2^ = 85.0%; *P* = 0.001) among the experimental groups. The individual negative predictive value in the experimental groups ranged from 19.5% (95% CI: 13.2% to 27.3%; Bretzinger et al., 2024, exp. 3) to 52.8% (95% CI: 38.6% to 66.7%; Bretzinger et al., 2024, exp. 2).

The objective of this meta-analysis was to evaluate the efficacy of AAM systems in identifying first ovulation and, consequently, the resumption of ovarian cyclicity in lactating dairy cows in early lactation. The majority of cows (88.1%) with estrous expression in early lactation ovulated based on blood P4. However, a large proportion of cows (74.7%) without estrous expression in early lactation ovulated as well (false negatives). Therefore, the results indicate that AAM systems were more effective at identifying ovulatory cows than anovulatory cows, as hypothesized. This is likely because of physiological aspects of the early postpartum estrous cycle rather than inaccuracies of AAM systems. The first ovulation postpartum is often “silent” or accompanied by weak estrous expression, followed by a short estrus interval (Crowe et al., 2014). The lack of robust estrous behavior may be because of high estradiol exposure during late gestation, inducing a refractory state that reduces the responsiveness of estrogen receptors (Allrich, 1994).

It is important to note that the low negative predictive value (35.3%) observed across studies may not solely reflect limitations of AAM systems or biological factors such as silent ovulations, but also the statistical influence of disease prevalence. Predictive values are known to be prevalence-dependent; specifically, negative predictive value decreases as the prevalence of the condition (in this case, cyclicity) increases (Altman and Bland, 1994). Given the high proportion of ovulatory cows in our dataset (79.8%), a lower negative predictive value is to be expected, even if the AAM system performs consistently in terms of sensitivity and specificity. Therefore, the low negative predictive value should be interpreted with caution, and future work could consider prevalence-adjusted modeling or the use of prevalence-independent metrics such as likelihood ratios to evaluate AAM performance more objectively.

Recent studies suggest that activity data within the VWP can aid in reproductive decision making (Borchardt et al., 2021; Bretzinger et al., 2023) and play a critical role in targeted reproductive management (Giordano et al., 2022). The relatively low negative predictive value of the AAM systems observed here should be considered for these types of management because cows without estrous expression in early lactation may be unnecessarily enrolled in complex timed AI protocols designed to improve fertility in anovular cows. For instance, supplementation of P4 during an Ovsynch protocol can improve P/AI in anovular cows, but not in cows with a corpus luteum (Bisinotto et al., 2015). Enrolling false negative cows in targeted protocols would increase costs without necessarily improving fertility outcomes. In addition to that, further research is warranted to compare reproductive outcomes in cows without estrous expression in early lactation based on their cyclicity status (i.e., cyclic vs. anovular) and whether these different subgroups show different benefits from enrolment into TAI protocols.

The overall prevalence of anovulation in the present study was 20.2%. This agrees with a US survey including 8 herds (mean 23.3%; ranging from 7.3% to 41.7%; Bamber et al., 2009) and a Canadian survey including 17 herds (mean 19.5%; ranging from 5.0% to 45.0%; Walsh et al., 2007) where anovulatory status was determined using serial blood or milk P4 measurements. There was, however, substantial variation among the experimental groups in our study ranging from 14.7% to 35.9%. The highest prevalence of anovulation was observed in the study with the shortest observation period (DIM 7–30; Borchardt et al., 2024). In addition to that, sampling frequency might have an impact on the detection of ovulatory status. Three out of 4 studies sampled cows every 2 wk (Bruinjé et al., 2023; Bretzinger et al., 2024; Laplacette et al., 2024). A sampling interval of 14 d may have limited the detection of short-lived luteal phases, particularly following the first postpartum ovulation, which is often followed by a short luteal lifespan. This constraint may have contributed to underestimating the true prevalence of cyclic cows. Such inconsistency among studies could reflect differences in the true proportion of anovulatory cows across herds or the different observational and sampling periods.

The overall prevalence of estrous expression within early lactation in the present study was 64.0%. There was, however, inconsistency among the experimental groups ranging from 27.6% to 89.5%. Studies that also used an AAM system to evaluate the prevalence of estrous expression in early lactation observed also a wide range across herds (Borchardt et al., 2021: 41.6%; Bretzinger et al., 2023: 79.2%). Based on differences in the observation period and AAM system used, results are difficult to compare.

The different AAM systems used across studies may differ in their sensitivity and specificity for detecting estrous and may introduce variability that cannot be accounted for, which is a limitation in this study. Additionally, variations in the timing and frequency of blood sampling for P4 measurements could have affected the estimation of true prevalence of onset of cyclicity. Future studies should involve multiple AAM systems installed on the same cows to obtain more accurate and comprehensive results. Inconsistencies in estrous expression across herds using different AAM system highlight the need for careful interpretation of sensor data because of varying test characteristics. Future research should aim to standardize methodologies, including uniform data collection periods and integrating multiple AAM systems within the same population, while evaluating the interaction between sensor-detected estrous behavior and metabolic or health parameters to improve predictive accuracy. Standardizing methodologies across studies will enhance comparability and improve the practical application of the AAM systems in reproductive management.

This meta-analysis reveals that AAM systems are promising tools in targeted reproductive management by accurately identifying ovulatory cows in early lactation. For instance, cows with an estrous alert during the VWP could be assigned to targeted programs designed for cyclic cows, which requires further investigation in different herds, geographical locations, and AAM systems. These targeted interventions in cows identified as ovulatory by AAM systems would likely involve protocols with reduced hormonal supplementation and greater reliance on spontaneous estrus detection by AAM for breeding decisions. Therefore, it would ultimately result in reproductive management approaches with reduced overall need of interventions and hormone use. As precision dairy technology advances, refining AAM systems to better detect anovulatory cows will be essential for maximizing their value in targeted reproductive management.
